# Association Between Herd Size and the Chemical Composition and Technological Properties of Milk Intended for Parmigiano Reggiano PDO Cheese

**DOI:** 10.3390/foods14030494

**Published:** 2025-02-04

**Authors:** Piero Franceschi, Paolo Formaggioni, Davide Barbanti, Yesid Orlando Gonzalez Torres, Cristina Scotti, Francesca Martuzzi

**Affiliations:** 1 Department of Food and Drug, University of Parma, Parco Area delle Scienze, 27/A, 43124 Parma, Italy; davide.barbanti@unipr.it (D.B.); francesca.martuzzi@unipr.it (F.M.); 2 Department of Veterinary Science, University of Parma, Via del Taglio 10, 43126 Parma, Italy; 3 Facultad de Ciencias Agrarias, Fundación Universitaria Juan de Castellanos, Cl. 17, #9-85, Tunja 150001, Boyacá, Colombia; ygonzalez@jdc.edu.co; 4 Centro Lattiero Caseario ed Agroalimentare, Strada dei Mercati 22, 43126 Parma, Italy; c.scotti@clca.it

**Keywords:** Parmigiano Reggiano cheese, milk production, herd size, milk composition, milk microbiological characteristics, milk technological characteristics, milk seasonal variations

## Abstract

The aim of this research was to compare the chemical composition and the technological characteristics of milk for Parmigiano Reggiano cheese produced in herds with different numbers of cows. The research was carried out on 5760 Italian Friesian herd milk samples collected from a total of 160 farms (one sample per month in each farm for three years). On each milk sample, lactose, fat, protein, casein, titratable acidity, total bacterial count, somatic cells, coliform bacteria, clostridia spores, and rennet coagulation properties were determined. Increasing herd size was positively correlated with milk production and with milk somatic cell and clostridia spores’ contents (8133 kg/cow/lactation, 5.280 Log_10_cells/mL and 1.782 spores/L for herds with less than 30 cows; 9109 kg/cow/lactation, 5.548 Log_10_cells/mL and 2.138 spores/L for herds with more than 200 cows, respectively). Moreover, herd size was negatively correlated with milk fat content and with total bacterial and coliform bacteria counts (3.73 g/100 g, 4.931 Log_10_CFU/mL and 3.176 Log_10_CFU/mL for herds with less than 30 cows; 3.51 g/100 g, 4.770 Log_10_CFU/mL and 3.121 Log_10_CFU/mL for herds with more than 200 cows, respectively). Farms with more than 100 cows raised were characterised by higher milk production per cow per lactation, but the milk produced by them was also characterised by lower fat content. Finally, milk produced in the herds with a higher number of cows showed a higher frequency of optimal lactodynamographic types (better rennet-coagulation properties) than milk produced in the other herds.

## 1. Introduction

Parmigiano Reggiano cheese is an Italian Protected Designation of Origin (PDO) product; it can be ripened from 12 to over 24 months and it is produced only with raw milk, calf rennet, and salt.

Furthermore, milk used in the Parmigiano Reggiano cheese-making process is produced by cows milked twice a day, at early morning and in the afternoon, and raised in farms placed within a restricted area reported by the PDO production regulation [1].

In the year 2023, the 292 dairies of Parmigiano Reggiano produced approximately 161 thousand tons of cheese, corresponding to about 2.02 million tons of milk (approximately 15.6% of all milk produced in Italy) from about 246 thousand cows raised in 2165 farms.

Moreover, these farms showed a wide variability in their size, considered as the number of cattle milked. Traditionally, small herds constituted the common farm type, but in recent decades, the number of cows per herd increased significantly [2]. This growth was related to the increase in the production cost, which reduces the profitability of the single animal, and by the necessity to increase the economic profitability of the farm [3].

It is important to consider that the structure and the management of small and large herds are radically different from each other. The first ones are typically tie-stall farms, where all operations, feeding included, are made by manual labour, and where milking is performed with milking machines by operators. On the other hand, the second type of herd is typically a free-stall farm, with automatization of herd operations, and feeding is frequently distributed by unifeed (total mixed ration), while the milking is made in milking parlour or by automatic milking systems.

Hence, all these characteristics can influence the animal’s welfare and consequently the production’s performance [4,5,6], with strong repercussions on milk chemical composition, physicochemical properties, and hygiene characteristics [4,7,8].

It should be highlighted that in the manufacture of Parmigiano Reggiano cheese, no additives may be used, and, in particular, the use of preservatives is strictly forbidden [1].

Moreover, since Parmigiano Reggiano cheese is produced with raw milk, a high level of total milk bacterial count, coliform bacteria, and clostridia spores may result in cheese structural, colours and sensory defects [9].

In addition, in the manufacture of Parmigiano Reggiano cheese, the cheese yield, like in other cheese types, is directly correlated to milk fat and casein contents [10,11], and, consequently, is influenced by parameters like somatic cell content [12,13].

It is important to underline that the milk’s chemical and microbiological composition and the technological properties are also influenced by other factors, like the environmental one [14]. Among these factors are the temperature and the humidity of the environment that are strongly affected by the seasons, which can be different between the years, and by the pedoclimatic zone [14,15,16,17,18].

However, the season is the most important factor affecting milk chemical and microbiological composition and milk technological properties. Indeed, several studies analysed the association between season and the variations in milk characteristics [14,17,18], and, among these, some of them directly investigated the changes in both chemical composition and microbiological characteristics of the milk destined for Parmigiano Reggiano cheese production [19,20].

Nevertheless, the climate changes are progressively influencing, during the years, the seasons, with the mitigation of the cold months and with the heating of the summer season [21].

This causes the increase in the heat stress of the cows, with negative repercussions on the milk’s technological properties [22].

For these reasons, the aim of this research was to compare the chemical composition, hygiene characteristics, and coagulation aptitude of milk for Parmigiano Reggiano cheese produced in herds characterised by a different number of cows.

Moreover, since it is important to monitor the effect of climate change on milk production, the secondary objective of this research is to study the effect of seasons on milk chemical composition and on milk physico-chemical properties.

Finally, the analysis of the principal components of the variance, to determine the main factors that affect the milk parameters variation, was performed.

## 2. Materials and Methods

### 2.1. Experimental Design and Sampling Procedure

Within the monthly sampling programme of the hazard analysis and critical control point (HACCP) system and of the milk quality payment system, in use in the production area of Parmigiano Reggiano PDO cheese, a total of 5760 herd milk samples were collected from 160 farms.

Among the 5760 herd milk samples, 1620 samples were collected in 45 farms with less than 30 cows in lactation; 1800 samples in 50 farms with from 31 to 60 cows in lactation; 1620 samples in 45 farms with from 61 to 100 cows in lactation; 540 samples in 15 farms with from 101 to 200 cows in lactation; and 180 samples in 5 farms with more than 200 cows in lactation.

Samples were collected during a period of 3 years, and samplings involved only mono-breed herds, raising only Italian Friesian cows. Moreover, herds management conditions were compliant with the Parmigiano Reggiano regulation: briefly, cows were milked two times a day, and they were fed only hay (at least 50% of dry matter of the diet), corn flour, and soybean germ [1].

The collection of samples was performed in farms located in the Parma province, directly from the cooling tank of the milk, at the end of morning milking, following the International Dairy Federation (IDF) standard [23]. After the sampling operation, the samples were cooled to 5 °C and transported immediately to the laboratory for analysis.

### 2.2. Analytical Methods

On each milk sample, the contents of lactose, fat, crude protein, and casein content, by mid-infrared spectrometry method with Milko-Scan FT+ (Foss Electric, DK-3400 Hillerød Denmark) were determined, according to the IDF standard [24].

On milk samples, the titratable acidity value was assessed by titration of 50 mL of milk with 0.25 N sodium hydroxide (Carlo Erba Reagents, 20007, Milan, Italy), according to the Soxhlet-Henkel method [25] with a Crison Compact Titrator D (Crison Instruments, 08328, Barcelona, Spain). Furthermore, on each milk sample, the total bacterial count, by flow cytometry method [26] with BactoScan FC (Foss Electric), and the somatic cell count, by fluoro-opto-electronic method [27] with Fossomatic FC (Foss Electric), were determined.

Moreover, the coliform bacterial count on Petri dish with Violet Red Bile Agar (VRB-agar; Merck KGaA, 64293, Darmstadt, Germany) ground after incubation at 37 °C for 24 h [28], and clostridia spores number by the method of the Most Probable Number (MPN) were also determined, as described by Franceschi et al. [29].

In brief, three aliquots of 10 mL, 1 mL and 0.1 mL of milk prepared with sterile ringer solution (Merck KGaA) were inoculated into five test tubes containing reinforced clostridial medium (RCM-OXOID; Thermo Fisher Scientific, 02451–02454, Waltham, MA, USA) with or without lactate.

Then, each tube was corked with 1.5 mL of mixture 1:1, wt/wt of paraffin and Vaseline (Carlo Erba Reagents) heated at 80 °C. Tubes were than incubated at 37 °C for 7 days and daily the raising of the cork due to the gas production was checked. The MPN counts were expressed as the number of spores per litres of milk. Using the MPN index of a special table, it is possible to predict the number of spores based on the combination of positive and negative test tubes.

The milk rennet coagulation properties, clotting time, curd firming time and curd firmness, were measured at 35 °C using Formagraph system of Foss Electric [30]. In this analysis, into 10 mL of milk were added 0.2 mL of rennet solution, obtained diluting 100 time a calf rennet with title of 1:100,000 (Chr. Hansen, I-20094 Corsico MI, Italy).

Then the samples were classified, starting from the values of clotting time, curd firming time and curd firmness, in 13 different lactodynamographyc types, identified with capital letters, according to Franceschi et al. [8]. After, these 13 types were grouped in 3 classes, always according to Franceschi et al. [8] (optimal, including A, B, C, EA, EB, EC types; discrete, including E, D, EF, DD types; poor, including FE, F, FF types).

Finally, from the dataset of Italian Breeders Association, for each herd, the quantity of milk produced from single cow per lactation was extracted, and the mean of milk production (kg/cow/lactation) for each herd was calculated.

### 2.3. Statistical Analysis

The Italian Breeders’ Association, basing on the Italian rural characteristics and the distribution of the number of cows raised on Italian farms, divides dairy cattle farms in five different classes.

Basing on this categorisation and according to Franceschi et al. [8], the herds were divided into 5 classes: less than 30 cows; from 31 to 60 cows, from 61 to 100 cows; from 101 to 200 cows and more than 200 cows.

Moreover, the values of the total bacterial counts, coliform bacteria count, the number of clostridia spores and the somatic cells counts were log transformed.

Data collected were tested by multivariate analysis of variance with software SPSS (IBM SPSS Statistics 29, Armonk, NY 10504-1722, USA), according to the following general linear model:

Y_ijklmn_ = µ + C_i_ + S_j_ + H_k_ + Z_l_ + V_m_ +ε_ijklmn_ (1)

where

Y_ijklmn_ = dependent variable; µ = overall mean; C_i_ = classes of herd size, 5 levels (i = 1, …, 5); S_l_ = effect of seasons (winter, from January to March; spring, from April to June; summer, from July to September; autumn, from October to December), 4 levels, (j = 1, …, 4); H_k_ = effect of housing types, 2 levels, tie stall or free stall (k = 1, 2); Z_l_ = effect of pedoclimatic zones of the herd, defined, in agreement with the territorial plan of Parma Province, in plain (up to 150 m above to the sea level) and hill (more than 150 m a.s.l.), (l = 1, 2); V_m_ = effect of years, 3 levels (m = 1, …, 3); ε_ijklmn_ = residual error

Moreover, the significance of the differences between classes of herd size and among the seasons were tested, always using the software SPSS, by the Bonferroni post hoc test.

In addition, the Pearson correlation coefficients were calculated between the data of the number of cows and milk production and composition. Furthermore, the correlations were considered weak with Pearson coefficient lower than 0.3, moderate with Pearson coefficient between 0.7 and 0.3, and strong with Pearson coefficient higher than 0.7 [16].

Furthermore, the differences among the distribution of lactodynamographic classes were tested with the chi-square method.

Moreover, data of number of cows, cow milk production, lactose, fat, crude protein, casein, somatic cells count, total bacterial count and coliform bacterial count were submitted to the principal component analysis (PCA) with software R 4.4.0 (R Foundation for Statistical Computing, Wien, 1020, Austria). In addition, the values of clostridia spores count and titratable acidity have not been considered in the principal components analysis of the variance because by a preliminary analysis of the data, they do not significantly influenced variance.

Furthermore, the independence of components was tested by the Bartlett Sphericity Test, and the components with the eigenvalues higher than 1 were extracted.

Finally, the matrix of the rotated component weights was obtained by the Varimax method.

## 3. Results

Differences among the herd size classes of milk production and milk chemical composition, physico-chemical properties, and microbiological characteristics are shown in Table 1; while Table 2 reports the Pearson correlation coefficients between the number of cows and milk production with milk composition parameters.

Milk production per lactation and milk contents of lactose, fat, crude protein, casein, and somatic cell count, total bacteria and clostridia spores were significantly different among herd size classes (*p* ≤ 0.001), while values of coliform bacteria showed significant differences among herd size classes with *p* ≤ 0.05.

Milk production per lactation progressively increased from the class with less than 30 cows to the class with more than 200 cows.

Lactose and crude protein were higher in the milk produced in the herd classes with over 61 cows with respect to the class with less than 30 cows, with intermediate values for the class from 31 to 60 cows.

Furthermore, milk casein content showed higher values in the milk corresponding to the classes with more than 61 cows with respect to the classes with less than 60 cows.

On the other hand, milk fat content was higher in the class with less than 30 cows and lower in the classes with more than 101 cows, with intermediate average values in the classes from 31 to 60 and from 61 to 100 cows.

Somatic cell count was lower in the classes with less than 30 cows and higher in the milk produced by the class with more than 200 cows, with intermediate average values in the other classes.

On the contrary, the average values of total bacterial count were higher in the herd in the classes with up to 100 cows than in the classes with more than 101 cows. A similar trend was observed for the coliform bacterial count. Indeed, for this microbiological parameter, values were higher in milk from the class with less than 30 cows and lower in milk from the class with more than 200 cows.

Finally, also clostridia spores showed significant differences among the classes (*p* ≤ 0.001) and their values progressively increased from the class with less than 30 cows to the class with more than 200 cows.

The herd size, expressed as the number of cows, resulted in a weakly positive correlation (*p* ≤ 0.001) with milk production (r = 0.247). Moreover, it was also weakly positively correlated (*p* ≤ 0.001) with milk titratable acidity, somatic cells count and clostridia spores count.

On the other hand, the number of cows was weakly negatively correlated (*p* ≤ 0.001) with milk fat content, total bacterial count and coliform bacteria count.

Furthermore, a weakly positive correlation between the number of cows and the milk contents of crude protein and casein was also recorded.

Finally, milk production was weakly positively correlated (*p* ≤ 0.001) with milk contents of lactose, crude protein, casein, titratable acidity and with somatic cell count, while it was weakly negatively correlated (*p* ≤ 0.001) with milk fat content and with total bacteria and coliform bacteria counts.

In Table 3, the results of chi-square test on lactodynamographic type classes are reported.

The number of samples with optimal coagulation properties and those with discrete coagulation properties showed differences among herd size classes (*p* ≤ 0.001).

In general, in all herd size classes, the number of samples with optimal coagulation properties was higher than that of the samples with discrete ones. In particular, the number of optimal lactodynamographic types was higher in the herds with more than 200 cows and lower in the herds with less than 30 cows.

On the other hand, the number of discrete lactodynamographic types was higher in the herds with less than 30 cows and lower in the herds with more than 200 cows.

Overall, the trends of the optimal and discrete lactodynamographic types showed opposite trends between them. The first increased with the increasing number of cows, while the second decreased.

In Table 4, the seasonal trends of the milk chemical composition, physico-chemical properties, and microbiological characteristics are reported.

All parameters presented in Table 4 showed significant differences among the seasons (*p* ≤ 0.001), except the coliform bacterial count, which showed a significant difference among the seasons with *p* ≤ 0.05.

The contents of lactose, fat, crude protein, casein and the titratable acidity showed lower values in summer and higher values in autumn and winter.

On the other hand, the average values of somatic cells, total bacterial, coliform bacteria and clostridia spore counts resulted higher in the milk of summer and lower in the milk produced in the winter season.

In Table 5, the eigenvalues of the variance components and their effect on the variance before and after rotation of the matrix values are reported, while in Table 6, the correlation values matrix of the rotated four principal components is reported.

Only the first four components of the variance showed eigenvalues higher than 1, and their cumulative variance after rotation was of 74.24%.

The first component of variance, with an eigenvalue of 2.06 after rotation of axis, accounts for 22.8% of the total variance and is strongly positively correlated with the milk contents of crude protein and casein.

Moreover, the second component of variance showed an eigenvalue of 1.67, that is lower if compared with the one of the first component and accounts for 18.51% of the total variance. Furthermore, the second component of the variance was strongly positively correlated with the lactose content and negatively correlated with the somatic cell count.

Nevertheless, the third component of variance, with an eigenvalue of 1.55, accounts for 17.27% of the total variance and was positively correlated with the number of cows and the milk production per lactation but was negatively correlated with milk fat content.

Furthermore, the fourth component of variance showed an eigenvalue of 1.40 and accounts for 15.58% of the total variance. It was positively correlated with the total bacterial count, and with the coliform bacterial count.

## 4. Discussion

Milk production per cow per lactation tendentially increases with the increasing of the number of cows raised on the farm. Indeed, milk production progressively increases from 8133 kg of milk per cow per lactation in herds with less than 30 cows to 9109 kg of milk per cow per lactation in herds with more than 200 cows. This observation is confirmed by the weak positive correlation between the two parameters.

Furthermore, milk lactose content significantly increases with the increasing of the number of cows raised on the farm. However, it should be noted that, although statistically significant, the variation in the milk lactose content among the herd classes results in a small entity. Indeed, the average values of milk lactose content ranged from 4.93 in the milk of herds with less than 30 cows to 4.97 g/100 g in the milk of herds with over than 200 cows. This observation is confirmed by the weak positive correlation between milk production and milk lactose content.

The association between the quantity of milk produced by the udder and the milk lactose content is confirmed by literature, as reported by Costa et al. [31], and it is due to the fact that lactose is synthesised in the mammary cells, starting with blood glucose [31,32].

In general, lactose, in association with minerals, keeps the equilibrium existing among the two parts of the basal epithelium that separates the blood from the milk and is the primary osmotic regulator between blood and alveolar lumen [33]. Indeed, the synthesis of lactose takes place in the Golgi, and after that, it is secreted by vesicles from the alveolar cells in the alveolar lumen [32]. Since lactose cannot pass through the alveolar membrane, this causes an increase in osmotic pressure in the alveolar lumen, and then water is absorbed by secretory cells to restore balance [33,34]. Therefore, the concentration of lactose determines the quantity of water absorbed in the alveolar lumen and, consequentially, the amount of milk produced [34].

In addition to milk lactose content, also the contents of crude protein and casein tendentially increase with the increasing of the number of cows raised on the farm. However, as happens for lactose, also the average values of protein and casein, despite being statistically significant, show variations among the herd classes of small entities.

The milk contents of crude protein and casein mainly depend on genetic factors; in the Parmigiano Reggiano area, during the last decades, cows have been intensively selected in order to continuously increase milk casein content. As a result of the increasing casein content, crude protein content also increased [8,35].

In general, beyond genetic factors, also the management can influence milk casein content. In particular, the feeding system is an important factor of variation, since it influences the rumen activity and affects the absorption of the nutrients and can limit the expression of the genetic potential, with repercussion on the production performance of the cows. Since the large-sized farms are more profitable than small ones, they can invest more economic resources in modern mechanical equipment for feeding cows [8]. It should be highlighted that, generally, large farms feed the cows with a total mixed-ratio diet, while small farms still administer the forage and the flour separately. Total mixed ratio diet, if it is correctly prepared and if it is properly administered to animals, facilitates the absorption of nutrients by the cattle and further the genetic potential expression [36,37].

On the other hand, milk fat content decreased with the increasing number of cows raised on the farm. This was confirmed by the weak negative correlation recorded between the number of cows raised on the farm and milk fat content.

The lower content of fat in the milk produced by herd classes with more than 100 cows is probably due to diet’s effect, as reported recently by Franceschi et al. [8]. In general, many studies, as, for example, Zebeli et al. [38], reported low fat content in milk produced by big modern farms, due to low-fibre content diets. Indeed, big modern farms are generally characterised by high production levels of milk and, also, by low milk fat contents, due to diets with high contents of starch and non-structural carbohydrates and low content of fibre [39,40,41].

It should be highlighted that, since cheese is constituted by a casein network that entraps fat globules, milk casein and fat contents have significant effects on cheese yield [10,42,43]. Milk casein and fat contents are important, also, in the milk transformation process into Parmigiano Reggiano cheese, because their milk contents strongly affect Parmigiano Reggiano cheese yield and, thus, the profitability of the milk [11].

Moreover, milk produced in herds that raise more than 100 cows in lactation resulted in less contamination, since it has a lower content of total bacterial count than milk from other groups. This is probably due to the relationship existing between the herd size and herd milk production level. Indeed, as confirmed by the positive correlation value, herds with more than 100 cows are characterised by higher milk production than the other herds. Franceschi et al. [8] reported that, since they are more profitable, herds with higher milk production can support greater investments, both in machinery and in organisational and management systems of the farm, to keep the level of milk pollution low.

Moreover, also the average values of coliform bacterial count resulted tendentially lower in the big herd with over than 200 cows, but in this case the difference among the average values is of very small entity and too much small to condition milk quality. Indeed, the average values of coliform bacterial count ranged from 3.18 Log_10_ (CFU/mL) in milk produced by herds with less than 30 cows to 3.12 Log_10_ (CFU/mL) in milk produced by herds with over 200 cows.

It should be highlighted that both values of total bacterial count and of coliform bacterial count (4.93 and 3.18 Log_10_CFU/mL, respectively) of milk produced in the herds with less than 30 cows are higher than those reported in the literature. For example, Franceschi et al. [29], in research carried out on herd milk obtained by an automatic milking system and by the traditional milking parlour in the Parmigiano Reggiano area, reported average values of total bacterial count and of coliform bacteria count of 4.29 and 2.16 Log_10_CFU/mL, respectively. On the other hand, the average value of total bacterial count (4.77 Log_10_CFU/mL) of the milk produced in the herds with more than 200 cows is in agreement with those observed by these authors [29].

Moreover, the average values of the total bacterial count of all classes resulted every time lower than the limit imposed by Regulation (EC) No 853/2004, which reports as a limit for the total microbial count of cow milk the value of 100 thousand CFU/mL, calculated as a rolling geometric average of the last two months with at least two samples per month [44].

Overall, in the production of raw milk cheeses, such as Parmigiano Reggiano, in general, total bacterial count is a very important parameter for the milk destined for the transformation. Indeed, a high value of total bacterial count produces, or simply increases, the risk of structural defects in cheese wheels and of alteration of colour and sensory properties of the cheese [9,45,46]. All these defects cause the devaluation of the cheese with high economic loss for the cheese-factory.

Since the production regulation of Parmigiano Reggiano cheese does not allow the use of preservatives, and since Parmigiano Reggiano cheese is produced with raw milk, also the presence in the milk of clostridia spores can cause several cheese defects during all the ripening processes of the Parmigiano Reggiano wheels.

In general, the method used in the Parmigiano Reggiano area to obtain the count of clostridia spores is the enumeration with the most probable number. In particular, the threshold of clostridia spores number, over which their content is considered high and milk is considered polluted by them, is 100 spores per litres [8,29]. From this point of view, the average value of milk clostridia spores of milk produced by farms with more than 200 cows (132 spore/L) can be considered high. While the average values of clostridia spores for herd milk produced by farms that raise more than 200 cows in lactation can be considered in agreement with that reported in the literature. On the contrary, the average values of clostridia spores for herd milk produced by farms that raise more than 200 cows must be considered in agreement with that reported by many authors in the last ten years. For example, Franceschi et al. [29] found an average value of 1.53 and 2.14 log_10_ (spores/L) for herd milk milked with automatic milking systems and herd milk milked in traditional milking parlours, respectively. Moreover, more recently, Franceschi et al. [8], in a study aimed at investigating the characteristics of milk for Parmigiano Reggiano produced in farms raising cows characterised by different milk production per lactation (from 6000 to 7999, from 8000 to 9999, and from 10,000 to 12,000 kg of milk per lactation), reported clostridia spores average values of 1.85, 1.85, and 1.80 Log_10_ (spores/L), respectively, for the three classes. Overall, these observations are consistent with the increasing trend of milk spore numbers recorded in the Parmigiano Reggiano area during the last decade by Franceschi et al. [8], and they confirm that clostridia spores remain one of the most diffuse problems for milk destined to Parmigiano Reggiano cheese.

Somatic cell counts average values ranged from 5.28 Log_10_ (cells/mL) in the milk produced by herds with less than 30 cows to 5.39 Log_10_ (cells/mL) in the milk produced by herds with more than 200 cows. This observation is confirmed by the positive correlation found between milk production and milk somatic cell count (r = 0.279; *p* ≤ 0.001). This trend could be related to the progressive increase in milk production that occurs when moving from small-sized farms to large-sized ones. This observation is in agreement with that reported by many studies. For example, Sadeghi-Sefidmazgi and Rayatdoost-Baghal [47] found a higher somatic cell count for milk produced in more productive farms (over 10,000 kg/305 days lactation).

Concerning milk somatic cell count, it is important to highlight that, since milk somatic cell count negatively affects milk casein content [12,48], decreasing it, and since it also negatively affects milk physico-chemical properties, such as milk acidity and milk rennet coagulation properties [12,13], they also negatively influence the cheese-making yield [10,43] and the cheese-making efficiency [16,42]. Franceschi et al. [13] reported a decrease in cheese-making yield in farm milk with over 400 thousand cells/mL.

Overall, the average values of the milk somatic cell count of all classes resulted under of this threshold.

In general, farms with more than 200 cows were characterised by a higher percentage of samples with optimal rennet-coagulation properties and a lower percentage of samples with discrete ones.

In particular, there is an increasing trend of the samples with optimal rennet coagulation properties and a decreasing trend of the samples with discrete rennet coagulation properties, moving from small-sized farms to large-sized ones. Since rennet coagulation properties of milk depend on many different factors, it is difficult to attribute these trends to a single specific factor.

In general, optimal rennet coagulation properties of milk are due to a high content of casein in milk [49], to a good amount and an optimal distribution of mineral salts and organic acids between the casein and the soluble phase, and to a suitable value of titratable acidity of the milk [42,49].

In general, in this case of study, tendentially, big herds showed higher casein content than small ones, and this could contribute to the higher rate of optimal coagulating samples.

The higher values of fat, crude protein, and casein in the milk produced in autumn than in that produced in spring are due to differences in the lactation stage of cows. Many authors generally confirm this. In particular, Franceschi et al. [50] reported that in the farms producing milk for Parmigiano Reggiano, most cows are in the early and late lactation stages, during the spring and the autumn seasons, respectively.

In general, the beginning of cow lactation is characterised by a fast increase in milk production, which is the main cause of the decrease in fat, protein, and casein contents [51,52]. Moreover, as lactation progresses, the milk production progressively decreases and milk fat, crude protein and casein contents progressively increase. Therefore, their content results higher in the late lactation stage than in the early [53,54,55].

Conversely, the contents of fat, crude protein, and casein in milk collected in the summer season, lower than those of autumn, are due to the hot-humid climate characterising this season [50]. Indeed, many studies on the Parmigiano Reggiano milk produced in the summer season reported a decrease in milk fat and crude protein contents and a general worsening of milk rennet coagulation properties [8,49]. This is due to the fact that in summer there are high temperatures with a high humidity index that cause heat stress to cows [19,20], which results in a reduction in milk yield [22], a reduction in milk fat and protein contents [49], and a worsening of the milk titratable acidity value that negatively affects the rennet coagulation properties [8,19,20].

In particular, Bernabucci et al. [20] recorded a lower fat content in summer milk (3.20 g/100 g) than in the milk produced in spring and in winter (3.61 and 3.80 g/100 g, respectively). This is confirmed also by the finding of Bertocchi et al. [19], who, in a study carried out on more than 508 thousand samples of herd milk, found in summer milk a fat content of 3.75 g/100 g and a value of 3.97 and 3.85 g/100 g in the milk produced in autumn and spring, respectively.

In addition, many studies investigated, also, the effect of heat stress of the cows on milk protein compounds. In particular, Cowley et al. [22] recorded lower casein content in milk produced by heat-stressed cows compared to the one produced by cows raised at the comfort temperatures (26.8 vs. 28.1 g/L, respectively). Furthermore, Bernabucci et al. [20] recorded a lower casein content in milk produced in summer (2.27 g/100 g) than in the milk produced both during the spring and the winter (2.48 and 2.75 g/100 g, respectively).

Overall, as reported by Cowley et al. [22], it seems that the heat stress can cause a feed intake reduction by cows, which results in a reduction by them of milk yield capacity and negatively affects milk fat, crude protein, and casein contents.

Furthermore, milk produced in the summer season is characterised by a higher somatic cell count, total bacterial count, and coliform bacteria than milk from all other seasons. Many authors confirm this. For example, Bernabucci et al. [20] recorded an increase in milk somatic cell content during the summer season. In addition, always, Bernabucci et al. [20] reported, also, that during the summer season there is a general increase in the values of total bacterial count and of coliform bacteria count.

The first component of variance resulted in a strong positive correlation with the milk content of protein and casein. This component very well describes the efforts of the farmers to improve herd genetic values by genetic selection and crossing plans. It is important to note that this component is the one that most affects the variance, because the increase in the milk protein content in general, and the content of casein in particular, is, together with the increase in milk production, the factor that mostly affects the profitability of the farm.

Moreover, the second component of variance was also found to be related to farm profitability. Indeed, this component resulted strongly positively correlated to milk somatic cell count and had a negative correlation to milk lactose content. This component of variance describes the mastitis and the problem of udder infections, very widespread in the intensive farms, such as those producing milk for Parmigiano Reggiano cheese.

Under this point of view, the response to the udder inflammation is associated with an increase in the somatic cells in milk [12,56], a crossing from the blood to the milk of some blood components [57], and a decrease in milk lactose content and a reduced milk production due to a general decrease in secretory activity of the mammary gland [12,58].

The third component of the variance describes the efforts of the farmers in the growth of their herd. Indeed, this component resulted in a strong positive correlation to milk production and to the number of cows raised in the herd. Also, this aspect was fundamental for the farm’s competitiveness and for its profitability.

Finally, the fourth component of the variance resulted in a strong correlation to the total milk bacterial count and the milk coliform bacterial count. This component describes the effort of farmers for the reduction in total bacterial count, due to the limit imposed by the Regulation (EC) No 853/2004 [44] that imposes a limitation of this parameter; and also describes the effort to avoid the contamination of milk.

## 5. Conclusions

In general, milk production per cow per lactation increases with the increasing of the number of cows raised on the farm.

Moreover, probably because they are more profitable, bigger farms can also develop new technologies aimed at reducing milk pollution. In particular, farms characterised by herds with more than 100 cows produce milk with a lower total bacterial count than the others.

On the other hand, in the bigger farms, associated with the increasing cow milk production, there is a decrease in milk fat content and an increase in somatic cell count, which can both negatively affect the milk profitability, as required by the milk quality payment system in use in the Parmigiano Reggiano cheese area.

Milk produced in the farms with more than 100 cows was also characterised by higher contents of clostridia spores; moreover, in the milk produced in the farms with more than 200 cows, its average value even exceeds the threshold limit of 100 spores/L.

Overall, since the profitability of a farm is strictly related to the level of milk production and since the milk production per cow per lactation increases with the increasing of the number of cows raised in the farm, the bigger farms are, the more competitive they are, compared to the others. This confirms the trend of the last thirty years, during which there was a progressive transition from a high number of small farms to a small number of big farms.

Finally, the diffused problem of milk pollution by clostridia spores, reported by many authors in the last ten years, is confirmed by our research.

Another confirmed result is that summer season is the period that more negatively affects milk quality. Climate change and global overheating probably will aggravate the heat-stress problems of dairy cattle; this, together with the effort to contrast the bacterial resistance to antibiotics, reducing the amount of antibiotics administered to the cattle, will be the two most urgent challenges to face in the immediate future.

## Figures and Tables

**Table 1:** Differences among the herd size classes of milk production and of chemical composition, physico-chemical properties, and microbiological characteristics of milk (least square mean values ± standard error).

		Less Than 30		From 31 to 60		From 61 to 100		From 101 to 200		Over 200		
		N ^1^ = 1620		N^1^ = 1800		N ^1^ = 1620		N ^1^ = 540		N ^1^ = 180		
Parameters	Measure Units	Mean	SEM ^2^	Mean	SEM ^2^	Mean	SEM ^2^	Mean	SEM ^2^	Mean	SEM ^2^	*p* ^3^
Milk production	kg/cows/lactation	8133	128 a	8275	129 a	8633	128 b	8877	134 c	9109	159 d	***
Lactose	g/100 g	4.93	0.01 a	4.95	0.01 ab	4.98	0.01 b	4.98	0.01 b	4.97	0.01 b	***
Fat	g/100 g	3.73	0.04 c	3.62	0.04 b	3.63	0.04 b	3.53	0.04 a	3.51	0.04 a	***
Crude protein	g/100 g	3.25	0.02 a	3.28	0.02 ab	3.30	0.02 b	3.29	0.02 b	3.29	0.02 b	***
Casein	g/100 g	2.42	0.01 a	2.52	0.01 b	2.58	0.01 c	2.56	0.02 c	2.57	0.02 c	***
Titratable acidity	°SH/50 mL	3.25	0.01	3.24	0.01	3.25	0.01	3.24	0.02	3.26	0.02	NS
Somatic cells	Log_10_ (Cells/mL)	5.28	0.02 a	5.36	0.03 b	5.38	0.02 b	5.39	0.03 b	5.55	0.03 c	***
Total bacterial count	Log_10_ (CFU/mL)	4.93	0.05 b	4.92	0.05 b	4.89	0.05 b	4.77	0.05 a	4.77	0.06 a	***
Coliform bacteria	Log_10_ (CFU/mL)	3.18	0.03 b	3.15	0.03 ab	3.16	0.03 ab	3.16	0.04 ab	3.12	0.04 a	*
Clostridia spores	Log_10_ (spores/L)	1.78	0.03 a	1.80	0.03 a	1.85	0.03 ab	1.91	0.03 b	2.14	0.04 c	***

^1^ Number of bulk milk samples. ^2^ Standard error of the mean. ^3^ Significance of differences obtained by analysis of variance: NS, *p* > 0.05; * *p* ≤ 0.05; *** *p* ≤ 0.001; a, b, c, d different for *p* ≤ 0.05.

**Table 2:** Pearson correlation coefficients between the number of cows and milk production with the milk composition parameters.

	Number of Cows Milked		Cow Milk Production	
	r ^1^	*p* ^2^	r ^1^	*p* ^2^
Cow milk production	0.247	***	-	-
Lactose	0.007	NS	0.228	***
Fat	−0.218	***	−0.362	***
Crude protein	0.033	*	0.064	***
Casein	0.032	*	0.076	***
Titratable acidity	0.058	***	0.123	***
Somatic cells count	0.097	***	0.279	***
Total bacterial count	−0.140	***	−0.187	***
Coliform bacteria	−0.056	***	−0.121	***
Clostridia spores	0.242	***	−0.041	**

^1^ Pearson correlation coefficient. ^2^ Significance of the coefficients compared to zero: NS, *p* > 0.05; * *p* ≤ 0.05; ** *p* ≤ 0.01; *** *p* ≤ 0.001.

**Table 3:** Differences among the herd size classes of the milk rennet coagulation aptitude (5760 bulk milk samples collected during 3 years from 160 herds).

	Less 30 N ^1^ = 1620			From 31 to 60 N ^1^ = 1800			From 61 to 100 N ^1^ = 1620			From 101 to 200 N ^1^ = 540			Over 200 N ^1^ = 180			
LDG ^2^	Count	%		Count	%		Count	%		Count	%		Count	%		*p* ^3^
Optimal	901	55.62	a	1064	59.11	b	965	59.57	b	352	65.19	c	127	70.56	d	***
Discrete	709	43.77	d	726	40.33	c	651	40.19	c	187	34.63	b	52	28.89	a	***
Poor	10	0.62		10	0.56		4	0.25		1	0.19		1	0.56		NS

^1^ Number of bulk milk samples. ^2^ Lactodynamographic types classes. ^3^ Significance of differences obtained by chi-square test: NS, *p* > 0.05; *** *p* ≤ 0.001; a, b, c, d different for *p* ≤ 0.05.

**Table 4:** Seasonal variations in chemical composition, physico-chemical properties, and microbiological characteristics of the 5760 bulk milk samples collected from the 160 herds.

Parameters	Measure Units	Winter N ^1^ = 1440		Spring N ^1^ = 1440		Summer N ^1^ = 1440		Autumn N ^1^ = 1440		SEM ^2^	Overall N ^1^ = 5760	*p* ^3^
Lactose	g/100 g	4.98	b	4.95	ab	4.90	a	4.96	b	0.04	4.95	***
Fat	g/100 g	3.64	b	3.52	ab	3.53	a	3.71	c	0.04	3.63	***
Crude protein	g/100 g	3.28	b	3.21	a	3.22	a	3.37	c	0.02	3.26	***
Casein	g/100 g	2.56	b	2.50	a	2.52	a	2.63	c	0.01	2.54	***
Titratable acidity	°SH/50 mL	3.26	b	3.25	b	3.23	a	3.26	b	0.02	3.24	***
Somatic cells	Log_10_ (Cells/mL)	5.35	a	5.38	b	5.46	c	5.38	b	0.03	5.40	***
Total bacterial count	Log_10_ (CFU/mL)	4.78	a	4.90	b	5.95	c	4.78	a	0.05	4.61	***
Coliform bacteria	Log_10_ (CFU/mL)	3.08	a	3.18	b	3.26	c	3.10	a	0.03	3.14	*
Clostridia spores	Log_10_ (spores/L)	1.88	a	1.88	a	1.92	b	1.91	ab	0.03	1.89	***

^1^ Number of bulk milk samples. ^2^ Standard error of the mean. ^3^ Significance of differences obtained by analysis of variance: * *p* ≤ 0.05; *** *p* ≤ 0.001; a, b, c different for *p* ≤ 0.05.

**Table 5:** Principal components analysis: eigenvalues of the components and their effect on the variance before and after rotation with Varimax method.

Component	Before Rotation Squares		After Rotation Squares		
	Eigenvalues	% of Variance	Eigenvalues	% of Variance	Cumulative %
1	2.20	24.45	2.06	22.88	22.88
2	1.91	21.26	1.67	18.51	41.39
3	1.39	15.43	1.55	17.27	58.66
4	1.18	13.11	1.40	15.58	74.24
5	0.76	8.44			
6	0.63	6.97			
7	0.50	6.07			
8	0.39	4.28			
9	5.27·10^−5^	5.86·10^−15^			

**Table 6:** Correlation values, after rotation, between four principal components of the variance and the number of cows, the milk production, and the milk characteristics.

	Components of Variance			
	1	2	3	4
Number of cows milked	0.078	0.243	0.668	−0.204
Cow milk production	0.075	−0.357	0.704	−0.120
Lactose	0.184	−0.832	0.043	−0.052
Fat	0.151	0.056	−0.768	−0.148
Crude protein	0.992	−0.011	−0.013	−0.032
Casein	0.994	−0.054	−0.010	−0.032
Somatic cells count	0.125	0.871	−0.025	0.154
Total bacterial count	−0.055	0.143	−0.135	0.778
Coliform bacteria count	0.000	0.048	0.037	0.831

## Data Availability

The data presented in this study are available on request from the corresponding author.
